# Supplementary material for: Health disparities and COVID-19: A retrospective study examining individual and community factors causing disproportionate COVID-19 outcomes in Cook County, Illinois
Source: PLoS One. 2022 May 16;17(5):e0268317. doi: 10.1371/journal.pone.0268317 (PMC9109922; doi:10.1371/journal.pone.0268317)
Supplement: Attachment — Submitted filename: KSOYEMI Comments to the Author 10-05-2021.docx [file pone.0268317.s001.docx]

**Health disparities and COVID-19: A retrospective study examining individual and**

**community factors and disproportionate COVID-19 outcomes in Cook County, Illinois,**

**March 16-May 31, 2020 Rebuttal to the Editor**

Dear Editors and Reviewers,

Thank you for your time and reviewing our manuscript. We appreciate your

feedback. All comments have been addressed. Please see our point-by-point responses as below.

Comments:
Abstract:
1a) (Lines 57-59): I would prefer that you write out IRR in the abstract, because it´s not a very often used abbreviation. p-Values differ in their number of digits, that should be changed.

***Response: IRR has been written out in the abstract as Incidence Rate Ratio and all the p-values have been rounded up to 2 digits.***

Introduction:
2a) (Lines 81-83): The wording is to strong for me. There is a rich body of scientific literature that links race/ethnicity to health outcomes. Maybe it´s more the awareness in society and the transmission of the scientific knowledge that is lacking. And in the next sentence you cite five papers linking social determinants and health outcomes.

***Response: Changes the wording to*** ***imply that while there is literature to support this assertion it is not widely acknowledged in society.***

2b) (Lines 96-100): Are these numbers confidence intervals and point estimates mixed? This part is confusing.

***Response: We clarified wording in the text.***

Materials and methods:
3a) (Line 116): Individual analysis -> Individual level analysis.

***Response:*** ***Changed Heading to “Individual level analysis”***

3b) (Lines 132-135): Percent below the federal povery line -> proportion of persons living below the federal povery line. The same for line 134.

***Response: Changed wording as suggested.***

3c) (Lines 165-172): You used a lot of model fit indices. Although I would not proceed as you did, I can agree on that. But the chosen model should not be part of the methods section. Move to results section.

***Response: In general, how the models are selected belongs to statistical method section. We agree to the reviewer that final chosen model can move to result section. Now we moved the chosen NB model to the beginning of results and keep other statistical methods in separate method section.***

3d) (Lines 173-176): You write that you compare comorbidities of different ethnic groups. It´s the lethality of Covid-19 in different comorbidity groups, right? Make the outcome clearer. ***Response: Clarifying language added to explain that these were comorbidities between decedents.***

3d) (Lines 177-178): Can you elaborate why this sentence stands there out of context after the statistical analysis?

***Response:*** ***Moved and added a sub-heading***

Results and discussion;
4a) (Lines 183-185): In Table 1 this number is 59%. Be consistent with your numbers. Mean is 73.8 and SD 14.73. The same number of digits should be used. The same applies for Line 185 and other parts of the text.
***Response: Corrected and rounded all percentages in the table. Overall, mean (SD) age at death was 74 (15) years. Hypertension was found to be the most frequent comorbidity and occurred in 6480 (60%) of the decedents, 4,461 (40%) had diabetes (Table 1).***

4b) (Table 1): The unknown category for cook county population by sex can be deleted as there are no unknown persons.

***Response: Deleted the unknown category under sex for table 1.***

4c) (Lines 193-195): You still write 777 of 1387 (56%), but in Table 2 you present a number of 37.7%. Giving two different percentages for the same number is very confusing. Table 2 would be so much easier to read, if you would present the 56% and do it accordingly for all values in the Table.

***Response: Corrected to say for both minorities and Whites, males recorded higher proportion of deaths 59% vs 56% respectively (Table 2).***

4d) (Line 210): Give an abbreviation for the effect measure you present here (IRR). Additionally, the numbers in the text and Table 3 are not the same.

***Response: We have corrected and checked all the numbers***

4e) (Lines 219-229): It is confusing to write 64% more likely and present an Odds Ratio of 1.37. How do you obtain the number 64% more likely? The same applies for the next sentences. Also, the interpretation of these results is not reflecting the Table very well, because you mainly interpret the Odds Ratios larger than 1. This whole part is very hard to read and confusing. I would recommend to exclude these lines and simply add columns to Table 2 and present the correct percentages for the comorbidities there.

***Response: We corrected and removed the percent now reads as Negative binomial regression showed that residents of Non-White ZCTAs were 1.02 times as likely to test positive and 1.15 times as likely to get a test when compared with residents of predominantly White ZCTAs [Incident Rate Ratio (IRR) 1.02, 95% CI (0.95, 1.10) and IRR 1.15, (0.99, 1.33)]. These rates were adjusted for population density, average household size, SDI score, per capita income, households living below the federal poverty line. When the number of deaths, positive tests, and number tested were modelled with no covariates, residents of Non-White ZCTAs were 1.67 times [IRR 1.62, 95% CI (1.23, 2.12)] more likely to die, 1.02 times as likely to be positive [IRR 1.02, 95% CI (0.81,1.02)] and 1.15 times as likely to be tested [IRR 1.15, 95% CI (0.99, 1.33)]. In the final model after adjusting for factors described above, residents of Non-White ZCTAs were 1.77 times as likely to die from COVID-19 [IRR 1.77, 95% CI (1.17, 2.66)].***

4f) (Lines 271-273): “56% or 1.4 more likely to die from Covid-19 …..(IRR=1.44)”. You confuse the reader by all this different numbers.

***Response: Corrected see above***

4g) (Lines 277-279): significantly higher chances (higher is missing). You reference to Table 3, that is not correct.

***Response: We have corrected and checked all numbers.***

4h) (Line 344): Delete to.

***Response: Corrected***

Reviewer #2: Thank you for the opportunity to review the revised version of Health Disparities and COVID-19: A Retrospective Study Examining Individual and Community Factors Causing Disproportionate COVID-19 Outcomes in Cook County, Illinois, March 16-May 31, 2020. The authors have generally addressed my comments, and the manuscript has improved. I am providing some additional feedback before submitting a publication recommendation.

• I appreciate the inclusion of social determinants of health. However, the introduction requires additional context regarding how the social determinants of health may contribute to health disparities.

***Response: We added the following: Half of deaths in the US are related to personal behaviors that are propelled by social factors, including income, education, and employment. Socioeconomic gradients are present among all health indicators.*** ***There is a causal link between social factors, health indicators, and measures of individuals’ socioeconomic resources, such as income, educational attainment, and social position. This association simulates a stepwise gradient pattern, with health improving incrementally as social position rises. This relationship is dose-dependent and is predominant in Non-Hispanic Black and Non-Hispanic White groups compared with Latinx populations. Social determinants of health contribute to disparities because differences that exist in life expectancy between racial and ethnic groups are socially and culturally established , not biologically derived. Long term exposure to stress induced by social and environmental factors cause biological “wear-and-tear” also known as allostatic load. Epigenetic processes that regulate whether genes are expressed or suppressed is one of the biological mechanisms involved in the linkage of social factors to health outcomes. Primate studies indicate that social status can affect the regulation of genes that control physiologic functions (e.g., immune functioning). Educational attainment, occupational class (e.g., manual vs. non-manual work), work schedules, stress, and intimate partner violence are linked with changes in DNA-protein complexes capping the ends of chromosomes (telomeres).***

• The third paragraph cites one study indicating that “differences in healthcare access and exposure risk” may contribute to Black and Latinx COVID-19 disparities, but there’s no explanation of why Black and Latinx persons experience these resource inequities. The social determinants of health, on which there’s a wealth of literature (see the cited Braveman manuscript), details these challenges. The current manuscript would benefit from a more careful application of the social determinants of health.

Response: we updated this section

***There is a causal link between social factors, health indicators, and measures of individuals’ socioeconomic resources, such as income, educational attainment, and social position. This association simulates a stepwise gradient pattern, with health improving incrementally as social position rises. This relationship is dose-dependent and is predominant in Non-Hispanic Black and Non-Hispanic White groups compared with Latinx populations. Social determinants of health contribute to disparities because differences that exist in life expectancy between racial and ethnic groups are socially and culturally established , not biologically derived. Long term exposure to stress induced by social and environmental factors cause biological “wear-and-tear” also known as allostatic load. Epigenetic processes that regulate whether genes are expressed or suppressed is one of the biological mechanisms involved in the linkage of social factors to health outcomes. Primate studies indicate that social status can affect the regulation of genes that control physiologic functions (e.g., immune functioning).***
• Line 85 – it’s unclear what ZCTA-level associations the authors are interested in. Poverty, household size?

***Response:We updated the analysis and updated the methods section .***

***We used the ZCTA Social Deprivation Index (SDI), a measure of ZCTA area level deprivation based on seven demographic characteristics collected in the American Community Survey (***[***https://www.census.gov/programs-surveys/acs/***](https://www.census.gov/programs-surveys/acs/)***). The SDI is used to quantify the socio-economic variation in health outcomes and levels of disadvantage across ZCTAs. The seven demographic characteristics are: percent living in poverty, percent with less than 12 years of education, percent single parent households, percent living in rented housing units, percent living in overcrowded housing units, percent of households without a car, and percent non-employed adults under age 65. We added average household size, average per capital income, percent of households that spoke English and Spanish, percent of households with bachelor’s degrees as additional covariates.***

Line 96 – please include the specific decedent demographics

***Response: We have corrected***

Variables used as indicators for the social determinants need to be explicitly detailed in the methods.

***Response:We have updated the methods section***

Lines 108 – 110 – please explain which variables are social determinants.

***Response: We have updated***

A rational for not including income in the models would be helpful. That is, average household size, poverty, income, etc. were obtained from 2018 ACS, yet income was not explored

***Response: We have addressed this by using the Social deprivation index***

The discussion section should be clearly labeled

***Response: Done***

Associations between several variables and COVID outcomes were explained in the discussion without making explicit connection to social determinants of health. I would like to recommend amending this section to reconnect with social determinants

***Response: We have addressed in the updated discussion***

As discussed, with poverty as the only indicator of SES, it should be accordingly be labeled poverty, not SES. For example, the authors argue that race is a stronger indicator of COVID-19 mortality than SES. Yet, according to their analyses, race is a stronger indicator than poverty.

Finally, there are still several typos.

***Response: We have corrected typos***

7. PLOS authors have the option to publish the peer review history of their article ([what does this mean?](https://nam12.safelinks.protection.outlook.com/?url=https%3A%2F%2Fjournals.plos.org%2Fplosone%2Fs%2Feditorial-and-peer-review-process%23loc-peer-review-history&data=04%7C01%7Clarissa.unruh%40cookcountyhhs.org%7Ca14e8e5b9d414e1c25b808d8e94b320b%7C3b922295e886417faaa84e4c4f069d82%7C0%7C0%7C637515856479017483%7CUnknown%7CTWFpbGZsb3d8eyJWIjoiMC4wLjAwMDAiLCJQIjoiV2luMzIiLCJBTiI6Ik1haWwiLCJXVCI6Mn0%3D%7C1000&sdata=hwjaW%2FC1RZqVi6YNIx3f0me9pm4stO20mSDo3T4m820%3D&reserved=0)). If published, this will include your full peer review and any attached files.


If you choose “no”, your identity will remain anonymous but your review may still be made public.


**Do you want your identity to be public for this peer review?** For information about this choice, including consent withdrawal, please see our [Privacy Policy](https://nam12.safelinks.protection.outlook.com/?url=https%3A%2F%2Fwww.plos.org%2Fprivacy-policy&data=04%7C01%7Clarissa.unruh%40cookcountyhhs.org%7Ca14e8e5b9d414e1c25b808d8e94b320b%7C3b922295e886417faaa84e4c4f069d82%7C0%7C0%7C637515856479017483%7CUnknown%7CTWFpbGZsb3d8eyJWIjoiMC4wLjAwMDAiLCJQIjoiV2luMzIiLCJBTiI6Ik1haWwiLCJXVCI6Mn0%3D%7C1000&sdata=7t1Jl0KqNytfYZskdduudaUzNZasiC4LqDQc5gIK4ss%3D&reserved=0).

Reviewer #1: No

Reviewer #2: No
