# Supplementary material for: Health disparities and COVID-19: A retrospective study examining individual and community factors causing disproportionate COVID-19 outcomes in Cook County, Illinois
Source: PLoS One. 2022 May 16;17(5):e0268317. doi: 10.1371/journal.pone.0268317 (PMC9109922; doi:10.1371/journal.pone.0268317)
Supplement: Attachment — Submitted filename: KSOYEMI Comments to the Author 04-10-2022.docx [file pone.0268317.s002.docx]

**Health disparities and COVID-19: A retrospective study examining individual and**

**community factors and disproportionate COVID-19 outcomes in Cook County, Illinois,**

**March 16-May 31, 2020 Rebuttal to the Editor**

Dear Editors and Reviewers,

Thank you for your time and consideration in reviewing our manuscript. We appreciate your

feedback. All comments have been addressed. Please see below for our responses.

Comments:

Introduction
Abstract:
1a) “There is a causal link between social factors, health indicators, and measures of individuals’ socioeconomic resources, such as income, educational attainment, and social position. This association simulates a stepwise gradient pattern, with health improving incrementally as social position rises. This relationship is dose dependent and is predominant in Non-Hispanic Black and Non-Hispanic White groups compared with Latinx populations.”

Comment: A reference for these sentences is necessary.

***Response:*** added citation [9] after that sentence

1b) “However, as is true with many diseases, COVID-19 outcomes are strongly associated with race.”

Comment: A reference is necessary.

***Response: added citations 13-17***

1c) “A systematic review by Mackay et al.”
Comment: After such a sentence the reference should appear. Additionally, this seems to be reference 9 (Mackey et al.).

***Response: added citation [18] after that sentence***

1d) “Similarly, same study showed that Latin X…”
Comment: English should be used properly.

***Response:*** added the word “the”

**Now reads :Similarly, the same study showed that Latin X**

Methods:
2a) per capital
Comment: per capita
***Response:*** removed the l from capital to make it per capita

Results:
3a) “Mean and standard deviation age at death was lower for Non-Whites compared with
Whites 70 (15) vs 78 (13); p < 0.01.“
Comment: Only the mean was lower.

***Response: removed “ and standard deviation” from this sentence***

3b) “risk difference of 0.03 [(95% CI: 0.15, 0.53); P = <0.01)]”
Comment: Firstly, a point estimate has to be part of the confidence interval. Secondly, you don´t write in the methods section that you calculated risk differences.

*“risk difference of 0.03 [(95% CI: 0.15, 0.53); P = <0.01)]”* *Firstly, a point estimate has to be part of the confidence interval.*
***Response: Corrected 0.03 (0.02,0.05)***

*Secondly, you don´t write in the methods section that you calculated risk differences.*

***Response: It was in the methods and was written as “We also reported the Relative Risk (RR) and risk differences and their corresponding 95% confidence intervals”.***

3c) “0.11 [(95% CI: -0.07, 0.30, 0.17); P = <0.20)] for hypertension“
Comment: There are three numbers in the confidence interval.

***Response: corrected to 0.11 [(95% CI: -0.07, 0.30); P = <0.20)] for hypertension***

Additionally, you report 60% of the non-white and 59% of the white decedents having a hypertension in Table 2. Thus, the risk difference is 0.01. There are more of these mistakes in Table 2.

***Corrected now reads 60% of the non-white and 59% of the white decedents had hypertension in Table 2. Thus, the risk difference is 0.01.***

3d) “0.04 [(95% CI: 0.03, 0.58); P = <0.01) for obesity.”
Comment: This confidence interval seems to be incorrect. Confidence intervals for risk differences are usually symmetric around the point estimate.

***Response: Corrected now reads 0.03 (0.02 ,0.05)***

3e) “Direct comparison of COVID-19 associated morbidities for Non-White compared with White decedents with hypertension showed little difference [Relative Risk (RR) 1.01, 95% CI
(0.98, 1.05)].”
Comment: If the confidence interval includes 1 they showed no difference.

***Response: changed “little” to “no”***

3f) “In the final model after adjusting for factors described above, residents of Non-White ZCTAs were 1.77 times as likely to die from COVID-19 [IRR 1.77, 95% CI (1.17, 2.66)]. Residents in households with a high percentage of Spanish speakers had increased risk of testing positive [IRR 1.69, 95% CI (1.03, 2.75)]. Living in poverty alone was not associated with an increased number of deaths, but the interaction of living in poverty and in a Non-White ZCTA increased the risk 2 to 3 times [IRR 2.99, 95% CI (0.71, 12.57)].”
Comment: In the previous version you didn´t include interactions. Although, the interaction is not significant, it seems relevant. But at least, tell the reader in the statistical analysis section that you investigated this interaction.

***Response:*** ***We added to the methods the following:*** ***For ZCTA analysis, we created one model for each outcome variable (mortality, number of positive tests and total number tested per ZCTA). For the mortality (deaths) per ZCTA, we used four models with sequential addition of economic and housing population covariates not included in the SDI. after each round of modelling To understand the relationships between the variables we added the interaction term of Non-White (Minority Zip) ZCTA and if greater than 20% of households that lived in poverty.***
Discussion:
4a) Comment: I don´t know about the exact guidelines of Plos One, but I find it uncommon to present new results (Figure 2) in the discussion section.

***Response: – moved the table out of discussion section***

4b) Comment: I think you cannot say COVID-19 infection. It should be COVID-19 or SARS-CoV-2-infection.

***Response:*** removed the word infection

4c) “As we compared comorbidities between ethnic/racial groups, we found that minority COVID-19 decedents had statistically significant chances of having comorbidities at the time of death.”
Comment: Significantly higher?

***Response: changed to*** “ higher likelihood of

Reviewer #3: WELL WRITTEN ARTICLE,
I ALSO VERY APPRECIATED THE CONCLUSION AND REFLECTION OF THE STRUCTURAL INEQUALITY WITH A CONSEQUENT DECREASE OF OPPORTUNITIES.

The following pertinent reports should be mentioned/discussed:

| PMID: 32374952 | Racial Health Disparities and Covid-19 - Caution and Context N Engl J Med 2020 Jul 16;383(3):201-203. doi: 10.1056/NEJMp2012910. Epub 2020 May 6. |
| --- | --- |
| PMID: 32293639 | COVID-19 and African Americans JAMA 2020 May 19;323(19):1891-1892. doi: 10.1001/jama.2020.6548 |
| PMID: 32305087 | COVID-19 exacerbating inequalities in the US Lancet 2020 Apr 18;395(10232):1243-1244. doi: 10.1016/S0140-6736(20)30893-X. |
| PMID: 35112280 | Utilization Gaps During the COVID-19 Pandemic: Racial and Ethnic Disparities in Telemedicine Uptake in Federally Qualified Health Center Clinic J Gen Intern Med 2022 Feb 2;1-7. doi: 10.1007/s11606-021-07304-4. Online ahead of print. |
| PMID: 35020749 | Increased mask adherence after important politician infected with COVID-19 PLoS One 2022 Jan 12;17(1):e0261398. doi: 10.1371/journal.pone.0261398. eCollection 2022. |
| PMID: 34997001 | Geographic and temporal variation in racial and ethnic disparities in SARS-CoV-2 positivity between February 2020 and August 2021 in the United States Sci Rep 2022 Jan 7;12(1):273. doi: 10.1038/s41598-021-03967-5. |
| PMID: 34836206 | l-Arginine and COVID-19: An Update Nutrients 2021 Nov 5;13(11):3951. doi: 10.3390/nu13113951. |
| PMID: 34762110 | Disparities in COVID-19 Outcomes by Race, Ethnicity, and Socioeconomic Status: A Systematic-Review and Meta-analysis JAMA Netw Open. 2021 Nov 1;4(11):e2134147. doi: 10.1001/jamanetworkopen.2021.34147. |
| PMID: 33519709 | Metformin Use Is Associated With Reduced Mortality in a Diverse Population With COVID-19 and DiabetesFront Endocrinol (Lausanne) . 2021 Jan 13;11:600439. doi: 10.3389/fendo.2020.600439. eCollection 2020 |
|  |  |

***Response: We replaced some of the previous references with the suggested references***
